# Endothelial Cell Glucose Metabolism and Angiogenesis

**DOI:** 10.3390/biomedicines9020147

**Published:** 2021-02-03

**Authors:** Wa Du, Lu Ren, Milton H. Hamblin, Yanbo Fan

**Affiliations:** 1 Department of Cancer Biology, University of Cincinnati College of Medicine, Cincinnati, OH 45267, USA; duwa@ucmail.uc.edu (W.D.); renln@ucmail.uc.edu (L.R.); 2 Department of Pharmacology, Tulane University School of Medicine, New Orleans, LA 70112, USA; mhamblin15@hotmail.com; 3 Department of Internal Medicine, Division of Cardiovascular Health and Diseases, University of Cincinnati College of Medicine, Cincinnati, OH 45267, USA

**Keywords:** endothelial cell, glycolysis, metabolism, angiogenesis, pathological angiogenesis, tumor microenvironment

## Abstract

Angiogenesis, a process of new blood vessel formation from the pre-existing vascular bed, is a critical event in various physiological and pathological settings. Over the last few years, the role of endothelial cell (EC) metabolism in angiogenesis has received considerable attention. Accumulating studies suggest that ECs rely on aerobic glycolysis, rather than the oxidative phosphorylation pathway, to produce ATP during angiogenesis. To date, numerous critical regulators of glucose metabolism, fatty acid oxidation, and glutamine metabolism have been identified to modulate the EC angiogenic switch and pathological angiogenesis. The unique glycolytic feature of ECs is critical for cell proliferation, migration, and responses to environmental changes. In this review, we provide an overview of recent EC glucose metabolism studies, particularly glycolysis, in quiescent and angiogenic ECs. We also summarize and discuss potential therapeutic strategies that take advantage of EC metabolism. The elucidation of metabolic regulation and the precise underlying mechanisms could facilitate drug development targeting EC metabolism to treat angiogenesis-related diseases.

## 1. Introduction

Vascular endothelial cells (ECs) form a single layer that coats the interior walls of arteries, veins, and capillaries. ECs are necessary for nutrient and oxygen exchanges between the bloodstream and surrounding tissues [1]. In response to proangiogenic stimuli, ECs rapidly change their cellular state from a quiescent to a proliferative and migratory state. Tip cells at the leading edge of sprouting vessel are characterized by a high migratory and matrix-degrading capacity. Stalk cells follow the tip cells, and they are highly proliferative. Compared with the two differentiated EC types, quiescent ECs are less proliferative and migrative [2]. Recently, metabolic pathways have been identified to be critical for many EC functions, including embryonic angiogenesis [3], pathological angiogenesis [4,5,6], inflammation [7], and barrier function [8]. By means of modern techniques, many new metabolic features of angiogenic ECs have been discovered. Increasing knowledge about the flexibility and adaptability of metabolism during the angiogenic switch will facilitate new therapeutic strategies for patients with angiogenesis-related diseases. Here, we shed light on the remarkable glycolytic features of angiogenic ECs and propose feasible therapeutic approaches targeting EC glucose metabolism.

### 1.1. Endothelial Cell Glucose Metabolism

For most body cells, carbohydrates, lipids, and proteins ultimately break down into glucose, fatty acids, and amino acids, respectively. The nutrients are used to produce energy through glycolysis or tricarboxylic acid cycle (TCA cycle) pathways. Glucose serves as the primary metabolic fuel to enter tissue cells and be converted to ATP for cellular maintenance. Thus, glucose metabolism is essential not only for energy production but also for metabolic waste removal. The endothelium is a metabolically active organ that maintains both vascular homeostasis and systemic metabolism [9]. In ECs, numerous genes, including insulin receptor substrate 2 (IRS2), peroxisome proliferator-activated receptor-gamma (PPARγ), and fatty acid translocase (FAT)/cluster of differentiation 36 (CD36), have been demonstrated to regulate systemic glucose levels [10,11,12,13,14]. Transcription factor EB (TFEB) is a master regulator of autophagy and lysosomal biogenesis [15]. Recently, we found that EC-specific TFEB overexpression improves systemic glucose tolerance in mice on a high-fat diet. In human primary ECs, TFEB increases glucose uptake and insulin transport across ECs through activation of Akt signaling [16]. It is still a challenge to elucidate detailed mechanisms by which altered EC function affects glucose metabolism in peripheral tissues in vivo. The role and mechanisms of these regulators in crosstalk between ECs and metabolically active tissues remain to be fully explored. 

In general, as the first step of glucose metabolism, glucose is transported across the plasma membrane by glucose transporters, especially glucose transporter 1 (GLUT1). As soon as glucose enters the cells, it is phosphorylated to glucose-6-phosphate (G6P) as catalyzed by hexokinase (HK). G6P can be utilized immediately for energy production through the glycolytic pathway. Under aerobic conditions, pyruvate can be fed into mitochondria and fully oxidized to produce ATP. When oxygen is limited, pyruvate is converted to lactate, and glycolysis becomes the primary source of ATP production [17]. In the early 1920s, Otto Warburg first found that tumor cells use the glycolysis pathway as the energy source under aerobic conditions. Until recently, ECs were discovered to have unique features of glucose metabolism [3]. Although glycolysis and oxidative phosphorylation (OXPHOS) are the two major energy-producing pathways in ECs, normally, up to 85% of ATPs are generated through the glycolysis pathway in human umbilical vein endothelial cells (HUVECs) during vessel sprouting [3]. In cultured quiescent aortic ECs, 99% of glucose is catalyzed into lactate [3,18]. In addition, single-cell RNA sequencing data obtained from mouse tumor tissues and choroidal neovasculature revealed that angiogenic ECs (tip, stalk cell) are enriched in gene sets of both glycolysis and OXPHOS when compared with normal ECs [4]. 

**Figure 1:**
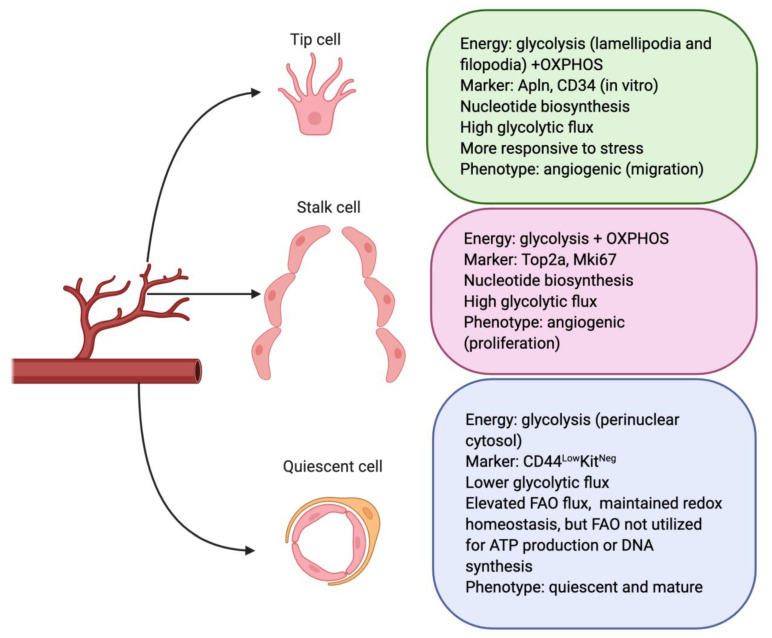
Differential metabolic features in three major endothelial cell (EC) populations. According to the phenotypes of ECs, they can be classified into tip cells, stalk cells, and quiescent cells during angiogenesis. Tip cells grow from the pre-existing vascular bed and are highly responsive to microenvironmental signals for migration. Stalk cells are highly proliferative and follow the tip cells to form a vessel lumen. Quiescent cells maintain vascular homeostasis. Angiogenic ECs show upregulated glycolysis gene signatures during the angiogenic switch to meet their metabolic demands. Quiescent ECs lower their glycolytic flux (35–40%) and use fatty acid oxidation (FAO) flux to maintain energy homeostasis. OXPHOS: oxidative phosphorylation.

ECs produce and consume energy to fuel cell proliferation and migration, maintain their structures, and adapt to environmental changes during the EC switch from a quiescent to an angiogenic phenotype. The different glycolytic features of tip cells, stalk cells, and quiescent cells are summarized in Figure 1. The unique aerobic glycolytic features of ECs could serve as beneficiary protection for ECs as follows: (1) glycolysis produces ATP with faster kinetics; (2) enabling rapid response to anaerobic conditions to generate energy, especially under nutrient deprivation; (3) glycolysis occurs in the cytosol and it does not require oxygen. ECs can save oxygen for the transendothelial transfer of oxygen to perivascular cells; (4) low mitochondria content (2–6%) in ECs is consistent with the role of mitochondria as an energetic sensor rather than producer [19]; (5) avoiding the production of reactive oxygen species (ROS) and preventing apoptotic cell death in oxidative stress conditions [20]; and (6) glycolysis provides metabolic intermediates to generate amino acids, lipids, and nucleotides [21]. In light of the importance of glycolysis in ECs, we summarize the role and underlying mechanisms of glycolysis in angiogenesis. 

### 1.2. Endothelial Cell Fatty Acid Oxidation

Fatty acids are another source of energy. They are either passively diffused from blood or transported by FAT/CD36 into the cell to fuel the TCA cycle [22]. Through the fatty acid oxidization (FAO) pathway, fatty acids are oxidized into two-carbon acetyl CoA molecules, which can provide twice as much ATP as carbohydrates. Strikingly, a recent study found that in cultured ECs, fatty acids act as a carbon source for deoxynucleoside triphosphate (dNTP) synthesis rather than providing ATP (<5% of total ATP) [23]. Genetic or pharmacological inhibition of carnitine palmitoyltransferase I (CPT1), the rate-limiting enzyme in FAO, causes functional defects in differentiation, proliferation, and barrier function in ECs [8,24,25]. Fatty acid transport protein (FATP) and fatty acid-binding protein (FABP) were also found to regulate EC function when stimulated with vascular endothelial growth factor (VEGF) [26,27]. Loss of fatty acid binding protein 4 (FABP4) in ECs results in decreased proliferation, migration, and sprouting [26]. Taken together, FAO is required for EC dNTP synthesis and proliferation during sprouting angiogenesis.

### 1.3. Endothelial Cell Glutamine Metabolism

Besides glycolysis and FAO, ECs utilize glutamine as a “conditionally essential” nutrient [28]. In physiological conditions, glutamine is the most abundant free amino acid in plasma and the most important donor of nitrogen atoms for metabolism [29], contributing 30% of the TCA carbons [23]. Nearly 90% of glutamine is transported into ECs through a sodium-dependent transporter system [30] and is oxidized in mitochondria to produce ATP. Highly proliferative ECs utilize glutamine for protein synthesis, the TCA cycle, and redox homeostasis [31,32]. Since ECs have low mitochondrial content, glutamine metabolism showed marginal effects on sprouting angiogenesis. Nevertheless, glutamine could be effectively regulating vascular tone and inflammation [32].

## 2. Glucose Metabolism in Quiescent ECs

In healthy adult vasculature, the majority of ECs are quiescent [33,34]. A quiescent endothelium is essential to maintaining vascular integrity, suppressing thrombosis, and inhibiting inflammation [34,35]. Laminar shear stress maintains the quiescent state of ECs, inhibits EC glycolysis, and downregulates PFKFB3 in a Krüppel-like factor 2 (KLF2)-dependent manner [36]. Forkhead box O1 (FOXO1), a transcription factor that plays an important role in regulating gluconeogenesis and glycogenolysis, maintains the EC quiescent state and restricts vascular overgrowth by reducing glycolysis. MYC proto-oncogene (MYC), a potent driver of anabolic metabolism, mediates the inhibitory effect of FOXO1 on glycolysis in ECs [37]. Unlike tip cells with compartmentalization of glycolysis in lamellipodia and filopodia, quiescent ECs have glycolysis taking place in the perinuclear cytosol [3]. ECs shift between a proliferative and nonproliferative state based on their metabolic needs. When angiogenesis occurs, EC migration and proliferation rely on glycolysis as a fuel source [38]. Quiescent ECs tend to have lower metabolic rates and reduced metabolic gene transcripts related to glycolysis, the TCA cycle, respiration, and nucleotide synthesis compared with highly activated ECs. Instead, through increasing FAO flux, quiescent ECs utilize FAO to maintain redox homeostasis but do not utilize FAO for ATP production or DNA synthesis [39]. This feature of metabolic adaptation and flexibility reprogram ECs to switch between angiogenic and quiescent states, which significantly impacts vascular disease-related angiogenesis.

## 3. EC Glucose Metabolism in Pathological Angiogenesis

### 3.1. Ocular Angiogenesis (Diabetic Retinopathy and Retinal Angiomatous Proliferation)

In diabetic patients, retinal ECs constitutively express GLUT1, which results in elevated ROS in both the cytosol and mitochondria due to high glucose level and insufficient ROS scavenging [40]. As a consequence, ECs lower down their glycolytic flux. Accumulated glycolytic intermediates are directed into other glycolysis branch pathways (e.g., ~3% enters the polyol pathway) and further increase ROS [41]. In the past few years, several studies were carried out to explore glucose metabolism in retinal ECs in the context of diabetic retinopathy. In vitro, loss of EC-*GLUT1* leads to reduced glycolysis, AMP-activated protein kinase (AMPK) activation, and decreased cell proliferation. Conditional deletion of *Glut1* in mouse ECs results in impaired retinal and brain angiogenesis due to tip cell reduction in vivo [42]. Deletion of peroxisome proliferator-activated receptor-alpha (PPARα) in endothelial colony-forming cells (ECFC) decreased mitochondrial oxidation and glycolysis and further exacerbated 4-hydroxynonenal (HNE)-induced mitochondria damage [43]. 

**Figure 2:**
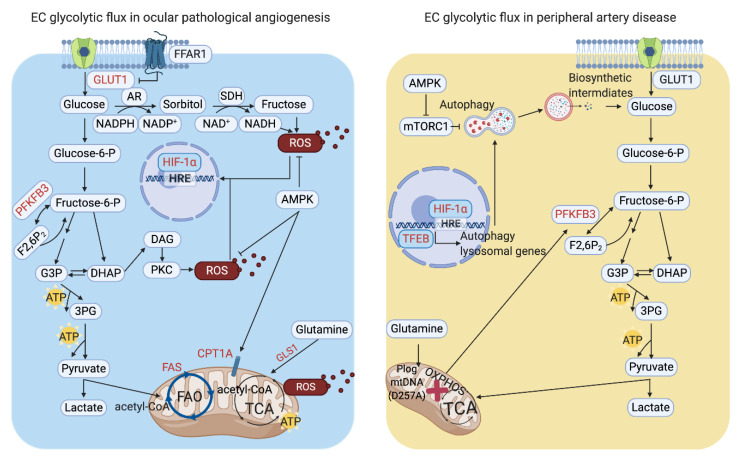
Endothelial cell (EC) glycolytic flux in ocular angiogenesis and peripheral artery disease (PAD). Retinal ECs utilize both glycolysis and fatty acid oxidation (FAO) for adenosine triphosphate (ATP) production. High glucose level leads to overproduction of mitochondrial reactive oxygen species (ROS) in ECs. The accumulated glycolytic intermediates result in lower glycolytic flux, which further increases ROS in both mitochondria and cytosol in diabetic ECs. ECs in PAD show impaired oxidative phosphorylation. Under energy deficiency conditions, phosphofructo-2-kinase/fructose-2,6-biphosphatase 3 (PFKFB3), glycolytic flux, and autophagy have protective effects on ECs in PAD. Red color represents the metabolic genes that positively regulate angiogenesis. GLUT1, glucose transporter 1; AR, aldose reductase; SDH, Sorbitol dehydrogenase; NADPH, nicotinamide adenine dinucleotide phosphate; NADH, nicotinamide adenine dinucleotide; AMPK, AMP-activated protein kinase; G3P, glyceraldehyde 3-phosphate; DHAP, dihydroxyacetone phosphate; DAG, diacylglycerol; PKC, protein kinase C; 3PG, 3-phosphoglyceric acid; FAS, fatty acid synthase; CPT1A, carnitine palmitoyltransferase 1A; GLS1, glutaminase 1; mTORC1, mammalian target of rapamycin complex 1; OXPHOS, oxidative phosphorylation; acetyl-CoA, acetyl coenzyme A; TCA, tricarboxylic acid cycle; F2,6P_2_, fructose 2,6-bisphosphate; TFEB, transcription factor EB; HIF-1α, hypoxia-inducible factor 1-alpha; HRE, hypoxia response elements; Plog, mitochondrial DNA polymerase gamma; mtDNA, mitochondrial DNA.

Recent studies of EC metabolic profiling shed light on the connection of EC metabolism to pathological ocular angiogenesis. Joyal et al. demonstrated that the retina utilized glucose and FAO for ATP production and identified that free fatty acid receptor 1 (FFAR1), a lipid sensor, inhibits glucose uptake when free fatty acids are available [44]. FFAR1 decreases GLUT1 and suppresses glucose uptake in the retinas of very low-density lipoprotein receptor (*Vldlr*) knockout mice. The impaired glucose uptake into photoreceptors results in a dual lipid/glucose fuel shortage and reduction in α-ketoglutarate, an intermediate of the TCA cycle. Low α-ketoglutarate further stabilized hypoxia-inducible factor 1α (HIF-1α) and increased VEGF secretion. As a result, abnormal vessels invaded avascular photoreceptors, which mimicked retinal angiomatous proliferation [44]. In addition, blockade of endothelial carnitine palmitoyltransferase 1A (CPT1A) or glutaminase 1 (GLS1) could reduce ocular neovascularization in mice [23,32]. Fatty acid synthesis is also involved in pathological ocular angiogenesis [45]. EC-specific fatty acid synthase (*Fasn*) knockout or application of the FASN blocker orlistat in vivo impairs angiogenesis and inhibits abnormal ocular neovascularization through malonylation-dependent repression of mammalian target of rapamycin complex 1 (mTORC1) activity. Taken together, the energy sources for ECs rely on both glycolysis and FAO in the pathological process of ocular angiogenesis, as shown in Figure 2.

### 3.2. Diabetic Angiogenesis

Hyperglycemia negatively regulates HIF-1α stability and its nuclear translocation by upregulation of prolyl hydroxylase domain protein 2 (PHD2) and PHD3. PHD2 and PHD3 act as oxygen sensors in oxygen-dependent regulation of HIF-1α stability. The hypoxia/VEGF axis is impaired in ECs under high-glucose conditions [46]. Under hypoxia, HIF-1α is also stabilized, and it upregulates glycolytic and glucose uptake-related genes, including glucose transporter 1/3 (GLUT1 and GLUT3), hexokinase 1/2 (HK1/2), phosphoglycerate kinase 1 (PGK1), lactate dehydrogenase A (LDHA), pyruvate kinase M2 (PKM2), phosphofructo-2-kinase/fructose-2,6-biphosphatase 3 (PFKFB3), aldolase A/C (ALDOA/C), glyceraldehyde-3-phosphate dehydrogenase (GAPDH), phosphofructokinase type 1 (PFK1), and pyruvate dehydrogenase kinase 1 (PDK1) [47]. Furthermore, in response to hypoxia, VEGF increases PFKFB3 expression to enhance glycolysis in ECs. However, high glucose reduces PFKFB3 expression in the mouse ECs [48]. These studies suggest that hyperglycemia downregulates two critical promoters of angiogenesis: HIF-1α and PFKFB3 in ECs. Therefore, different from diabetic retinopathy, diabetes leads to insufficient angiogenesis in wound healing [49,50,51], characterized by decreased angiogenesis and vascular density.

In diabetic rodents and humans, peroxisome proliferator-activated receptor-gamma coactivator 1-α (PGC-1α) expression was elevated in ECs [52]. PGC-1α could be a critical regulator of endothelial activation caused by hyperglycemia [52]. In mice, endothelial PGC-1α inhibits blood flow recovery, exacerbates foot necrosis in the hindlimb ischemia model, and attenuates wound healing. Mechanistically, PGC-1α activates Notch and blocks Rac/Akt/eNOS signaling in ECs [52]. Collectively, as critical metabolic regulators in ECs, PFKFB3, HIF-1α, and PGC-1α could be potential targets to modulate angiogenesis in diabetic conditions.

### 3.3. Peripheral Arterial Disease (PAD) and EC Glycolytic Flux

PAD is a manifestation of reduced blood supply to the lower extremities that is mostly induced by atherosclerotic obstruction. Ischemia imposes a major energetic challenge on the tissues due to impaired oxidative phosphorylation. Using Polg mtDNA mutator (*Polg^D257A^*) mice, Ryan et al. observed the remarkable protective effects of glycolytic metabolism and PFKFB3 on hindlimb ischemia. They also collected muscles from patients with critical limb ischemia and found lower PFKFB3 expression and reduced glycolytic flux in patient muscles [53]. Indeed, therapeutic angiogenesis is a promising strategy for the treatment of PAD. Utilizing EC-specific *Tfeb* transgenic and knockout mice, we demonstrated that TFEB promotes angiogenesis and improves blood flow recovery in the mouse hindlimb ischemia model. In ECs, TFEB increases angiogenesis through the activation of AMPKα and upregulation of autophagy [54,55,56]. As summarized in Figure 2, this finding established a positive relation between TFEB and postischemia angiogenesis. The role of TFEB in glucose metabolism remains to be fully explored.

### 3.4. Tumor Angiogenesis

#### 3.4.1. Tumor Endothelial Cells (TECs) Adapt Their Metabolism to the Tumor Hypoxic Environment

**Figure 3:**
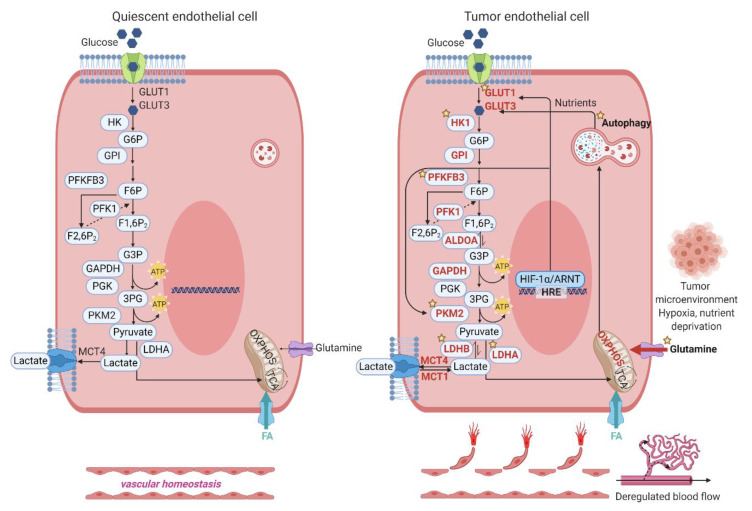
Tumor endothelial cells (TECs) exhibit distinct metabolic transcriptome signatures, which are linked to their angiogenic potential. Compared with quiescent ECs, TECs utilize both glycolysis and OXPHOS for energy production. TECs (tip, stalk, and immature ECs) show upregulated glycolytic genes, including glucose transporter 1 (GLUT1), glucose transporter 3 (GLUT3), hexokinase 1 (HK1), hexokinase 2 (HK2), 6-phosphofructo-2-kinase/fructose-2,6-biphosphatase 3 (PFKFB3), phosphofructokinase 1 (PFK1), aldolase A (ALDOA), glyceraldehyde 3-phosphate dehydrogenase (GAPDH), pyruvate kinase M2 (PKM2), enolase 1 (ENO1), lactate dehydrogenase A (LDHA). TECs can proliferate in a lactate-rich environment. Under hypoxia, hypoxia-inducible factor-1 alpha (HIF-1α) increases the expression of GLUT1 and GLUT3 in TECs. Autophagy is increased to promote TECs to survive and adapt to metabolic needs. The star symbol indicates the steps where chemical compounds are available and the antiangiogenic effects have been tested in preclinical or clinical settings. Red color represents the upregulated metabolic genes. FA, fatty acid; MCT1/4, monocarboxylate transporter 1/4; PGK, phosphoglycerate kinase; GPI, glucose-6-phosphate isomerase.

ECs are more resistant to hypoxia than other cell types, such as cardiomyocytes and neurons [57]. Unlike normal ECs, disorganized TECs are essential for tumor growth characterized by a leaking vascular system, high interstitial fluid pressure, reduced blood flow, poor oxygenation, and acidosis [58]. Hypoxia and ischemia can activate the EC switch from a quiescent to angiogenic phenotype (higher proliferative and migratory abilities). Recently, single-cell RNA sequencing data from tumor tissues revealed that TECs are hyperglycolytic. Their total read counts were 2–4 fold higher than that in normal ECs, which means TECs have high RNA content due to increased metabolic demands of nucleotide biosynthesis and glycolysis [4]. TECs have a distinct metabolic transcriptome signature linked to their angiogenic potential, as shown in Figure 3. Similar to TECs, stroma cells also have metabolic adaptability or flexibility in the tumor microenvironment [59]. TECs showed heterogeneity in cell function and structure, which could be varied in different host organs and tumor types [4,60]. To date, although many antiangiogenic compounds have been identified, antiangiogenesis therapy may not be enough for tumor treatment (metastatic breast cancer and glioma) due to vessel co-option or vasculogenic mimicry [61,62]. Since tumor cells and tumor stroma cells, including TECs, can adapt their metabolism to survive and proliferate in tumor growth, modulation of cell metabolism could be effective to control not only different phenotypes of TECs at multiple steps of angiogenesis (proliferation, migration, sprouting, and maturation) but also tumor cell growth. 

#### 3.4.2. TECs Release More Lactate and Utilize Lactate for Proliferation

Lactate, the metabolic end-product of glycolysis, is released by ECs in aerobic conditions, and in turn, lactate attenuates HIF-1α degradation by binding and inhibiting HIF prolyl hydroxylase [63]. Hyperglycolytic TECs could be the potential source of lactate in the tumor microenvironment. Both normal ECs and TECs can utilize lactate to support their growth in a dose-dependent manner [63]. In the mouse Lewis lung carcinoma model, suppression of monocarboxylate transporter 1 (MCT1), the main transporter for lactate uptake in ECs, reduces tumor angiogenesis [64]. Lactate dehydrogenase B (LDHB) expression is increased in TECs, which helps re-entered lactate to integrate into the metabolism [65]. Compared with normal ECs, TECs do not only produce more lactate; their growth is also promoted by lactate preferentially. Unlike normal ECs, TECs can proliferate in a lactate-rich environment due to highly expressed carbonic anhydrases II (CAII) that facilitate the transport activity of MCT1/4 [58]. These findings suggest that TECs are more glycolytic than normal ECs, even though normal ECs are already addicted to glycolysis [3]. Collectively, altered TEC glucose metabolism can sustain their proliferation in the tumor microenvironment and survive in an acidic environment.

#### 3.4.3. TECs Display High Glycolytic Flux

Accumulated single-cell RNA sequencing data has revealed numerous transcriptome signatures related to glycolysis in TECs [4]. Compared with normal ECs, TECs showed higher glycolytic flux. The mechanisms by which glycolytic flux is regulated in TECs remain to be fully explored. The first key and irreversible step is the transformation of F6P to fructose-1,6-bisphosphate (F1,6BP) catalyzed by phosphofructokinase 1 (PFK1). PFK1 activity is inhibited by intracellular ATP or citric acid and reactivated by F2,6BP. PFKFB3 has high kinase activity to promote the synthesis of F2,6BP and maintain the increased glycolytic flux [66]. EC-specific deletion of a single *Pfkfb3* allele or administration of the PFKFB3 inhibitor (3-(3-pyridinyl)-1-(4-pyridynyl)-2-propen-1-one, 3PO) reduces tumor cell invasion and metastasis, normalizes tumor vessels, and improves the vascular barrier by reducing VE-cadherin (vascular endothelial cadherin) endocytosis [5]. Augmented glycolysis in TECs fuels multiple metabolic pathways, including the pentose phosphate pathway (PPP), hexosamine biosynthesis pathway (HBP), TCA cycle, and serine biosynthesis pathway [4]. Glycolytic flux is nearly three-fold higher in TECs than in normal ECs, and TECs utilize glucose carbons for biomass production. Additionally, in TECs, hypoxia upregulates glucose transporters (GLUT1 and GLUT3), which are necessary for rapid glucose uptake and increased glycolytic flux [5,67]. Both inter- and intratumor metabolic heterogeneity has been observed within and between the tumors [68]. This would make the strategy of targeting glucose metabolism in TECs more valuable, as tumor cells show high metabolic flexibility. At the same time, TECs are more stable and consistent among various tumor types. 

#### 3.4.4. TECs Exhibit Increased Autophagy

**Figure 4:**
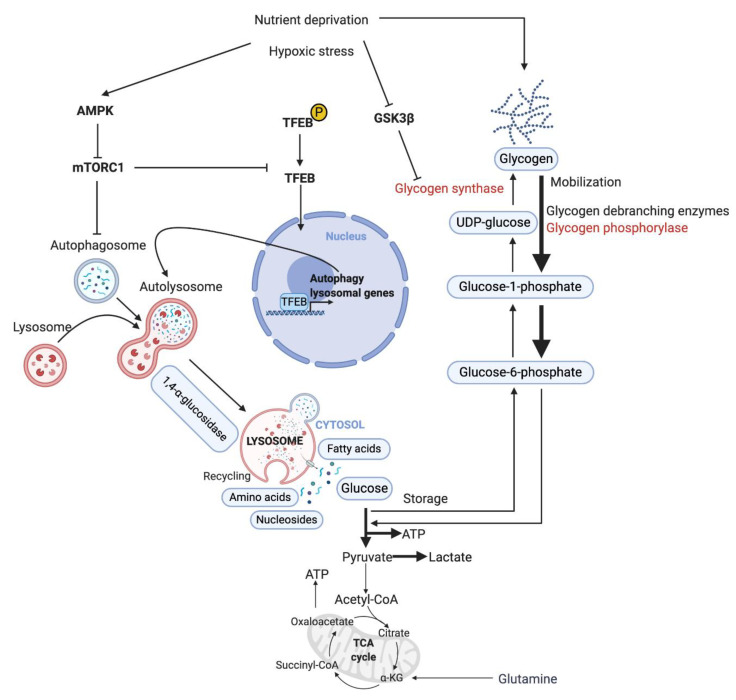
EC glucose and glycogen metabolism in hypoxia and nutrient-deprivation conditions. Under hypoxia and nutrient-deprivation conditions, cellular energy levels are decreased and autophagy is upregulated by the AMPK–mTORC1 pathway. The autophagy–lysosomal pathway promotes the recycling of nutrients, including glucose, for cell survival. In response to environmental changes, ECs use glycogen as a backup energy source. Upon nutrient deprivation, TFEB translocates to the cell nucleus, where it activates target genes involved in lysosomal function and autophagy. Upregulated autophagy/lysosomal activity supports ECs to resist the detrimental effects of hypoxia and nutrient deprivation. Red color represents the key metabolic genes.

Autophagy is a conserved cellular degradation pathway that is critical to maintain cellular homeostasis and to adapt to the metabolic needs to sustain proliferation and survival [69,70]. However, the effects of autophagy in cancer are still controversial because autophagy plays opposite roles in precancerous and malignant tumors [71,72]. The role of autophagy in the vasculature has gained more attention as tumor vessels are involved in both nutrient replenishment and metastasis for starved and stressed tumor cells. In the tumor microenvironment, TECs are subjected to low glucose, starvation, low blood flow, and hypoxia. Autophagy is upregulated in TECs in response to extracellular stresses [69]. Mechanistically, autophagy is controlled by upstream regulators, including mammalian target of rapamycin (mTOR) and AMP-activated protein kinase-α (AMPKα). Accumulated studies suggest that autophagy regulators, including Beclin 1 (BECN1), TFEB, and high-mobility group box protein 1 (HMGB1) modulate angiogenesis [54,65,73]. Compared with normal ECs, TECs may adjust their autophagy/lysosomal activity to mitigate the detrimental effects of hypoxia. ECs maintain glycogen stores during hypoxia but not under low-glucose conditions. Autophagy sustains cell survival in nutrient-deprivation conditions, in which cells use glycogen as a critical backup energy source [74]. In Figure 4, we summarize the glycogen storage and breakdown pathways modulated by autophagy. In a high-glucose environment, ECs store glycogen to prepare energy for extracellular stresses. Upon hypoxia or nutrient deprivation, 90% of glycogen is mobilized and converted into glucose-1-phosphate (GP) catalyzed by glycogen debranching enzymes and glycogen phosphorylase [75]. Then, GP is converted into glucose-6-phosphate (G6P) catalyzed by phosphoglucose mutase. G6P can be directly used for glycolysis to maintain EC proliferation and migration. Alternative autophagy-dependent glycogen breakdown (10%) produces nonphosphorylated glucose catalyzed by lysosomal 1,4-α-glucosidase to meet metabolic requirements [76]. Nonphosphorylated glucose can either be used in glycolysis or stored as glycogen in cells, depending on the metabolic status. In various cell types, TFEB increases autolysosome numbers and stimulates the fusion between lysosomes and autophagosomes under hypoxia and nutrient-deprivation stress [77,78]. In this scenario, TFEB, together with other autophagy regulators, would be critical in glycogen storage and mobilization through enhancing autophagic flux. The role of autophagy in TEC metabolic reprogramming remains to be fully explored.

## 4. EC Metabolic Regulators of Antiangiogenesis and Vessel Normalization

In both preclinical and clinical settings, anti-VEGF therapy showed transitory, limited efficacy and acquired resistance [79]. Recent single-cell RNA sequencing data suggested that glycolytic genes were upregulated in tip cells in xenograft tumors after pharmacological inhibition of VEGF and Notch signaling [60]. Abnormal tumor vessels promote tumor growth, metastasis, and resistance to chemotherapy. Tumor vessel normalization has been recognized as a promising strategy for anticancer treatment. Blockade of PFKFB3 improves vessel maturation and perfusion, thereby reducing tumor cell invasion, intravasation, and metastasis and enhancing the efficiency of chemotherapy on tumors [5]. Glycolysis drives EC rearrangement by increasing filopodia formation and reducing intercellular adhesion. PFKFB3 blockade promotes the disturbed EC rearrangement in high-VEGF conditions [80]. The glycolytic enzyme pyruvate kinase M2 (PKM2) regulates cell–cell junctions and migration in ECs. PKM2 knockdown promotes proper VE-cadherin internalization/traffic at endothelial junctions, which may help vessel normalization in tumors [81]. Thus, manipulation of TEC glycolysis for vessel normalization constitutes a potential therapeutic intervention in tumors.

Taken together, targeting TEC glucose metabolism and thereby inhibiting angiogenesis is a promising strategy for cancer treatment. We summarize the compounds that target critical metabolic enzymes in glycolysis and other metabolic pathways in Table 1. Of note, these compounds regulate metabolism in both TECs and cancer cells. For instance, cancer cells also readily use glycolysis for energy metabolism [82]. Therefore, understanding TECs in metabolism and antiangiogenic resistance can help develop novel strategies to treat cancer.

## 5. Conclusions and Open Questions

The metabolic regulation of ECs is gaining much attention, especially in pathological angiogenesis within the tissue-specific microenvironment. EC metabolism has been summarized into glucose metabolism, fatty acid oxidation, and glutamine metabolism. In this review, we summarize glucose metabolism within quiescent and angiogenic ECs. Metabolic adaption of ECs during the angiogenic switch and in healthy tissues is well-documented [116]. TEC metabolic reprogramming should be studied as a common and expected feature of metabolism. Many antiangiogenic therapies are designed to inhibit VEGF receptors or VEGF signaling in ECs, which results in the insufficient treatment of tumors and increased tumor metastasis due to elevated hypoxia in the tumor microenvironment [117]. It could be a promising strategy to modulate metabolic regulators in TECs. Targeting TEC metabolism would allow us to design new strategies combined with the classical antiangiogenic strategies to fight cancer.

Glycolysis, but not oxidative phosphorylation (OXPHOS), is chosen for ATP generation in cultured human primary ECs, mouse angiosarcoma, and mouse hemangioma [3]. However, single-cell RNA sequencing data from in vivo tumor tissues suggested that angiogenic ECs (tip cells, proliferating cells, and immature cells) still rely much on both glycolysis and OXPHOS as energy sources [4]. During tumor blood vessel sprouting, whether large amounts of glucose are available is still speculative. Since aerobic glycolysis is upregulated in TECs, they should be classified as oxidative ECs and glycolytic ECs, even within the same tumor. This would be beneficial for further understanding of the metabolic demands in tumor bioenergetics. TECs support tumor progression and affect chemotherapeutic resistance and metastasis. The metabolic switch is not specific to ECs but exists as an example of global adaptation and flexibility to environmental changes. 

Unlike tumor cells that carry various mutations, TECs are more genetically stable. Targeting the metabolism of TECs instead of tumor cell metabolism could be a promising strategy against tumor progression. It is expected that metabolic modulators are able to affect different steps of the angiogenic process in ECs. Here, we summarize some questions that remain to be answered: What is the exact role of the autophagy/lysosome pathway in EC metabolism? Is there any glucose competition between ECs and other cell types, including tumor cells, stromal cells, and macrophages, in the tumor microenvironment? How do TECs escape from cell death in hypoxic and nutrient-deprived conditions? Understanding the role and underlying mechanisms of EC metabolism will facilitate new therapeutic approaches to modulate angiogenesis-related diseases.

## Figures and Tables

**Table 1:** Compounds that directly target metabolic enzymes involved in tumor angiogenesis.

Target	Compound	Tumor Type	Status
**Glycolysis**			
Glucose transporters Glut1	Phloretin Silybin (Silibinin) Canagliflozin Curcumin Fasentin Genistein	Cervical cancer cell Prostate cancer Liver cancer cell Multiple cancers Breast cancer Multiple cancers	Preclinical [83] Clinical phase II [84] Preclinical [85] Clinical phase I/II [86] Preclinical [87] Clinical phase I/II [88,89]
Hexokinases	2-DG ^1^ Ketoconazole Lonidamine Methyl jasmonate	Multiple cancers Glioblastoma Melanoma, breast cancer, glioblastoma, lung cancer, prostate cancer Gastric cancer	Clinical phase I/II [90,91,92] Preclinical [93] Clinical phase III [94] Preclinical [95]
PFKFB3 ^2^	3PO ^3^ PFK158	Melanoma, lung carcinoma, pancreatic cancer Advanced solid malignancies	Preclinical [5,96,97] Clinical phase I [98]
Pyruvate kinase-M2 (PK-M2)	TLN-232 Shikonin	Metastatic renal cell Lung carcinoma	Clinical I/II [99] Preclinical [100]
Lactate dehydrogenase	PTK787/ZK 222584 (Vatalanib) Gossypol Oxamate	Colon cancer, advanced colorectal cancer Multiple cancers Breast cancer	Preclinical [101] Clinical phase I/II [102,103] Preclinical [104]
Lactate	Lonidamine AZD3965	Prostate cancer Gastric cancer, prostate cancer lymphoma	Clinical phase III [94] Clinical phase I [105]
**TCA cycle**			
PDK1 ^4^	Dichloroacetate (DCA)	Non-small-cell lung cancer, breast cancer	Preclinical [106]
**OXPHOS**			
Mitochondrial complex I/III	Metformin Phenformin Arsenic trioxide	Breast cancer Cholangiocarcinoma Gastric cancer cells	Preclinical [107] Clinical phase I [108,109] Preclinical [110]
**FAO**			
CPT1 ^5^	Etomoxir Perhexiline	Lung carcinoma, prostate cancer cell line Prostate cancer, glioma	Preclinical [23,111,112] Preclinical [112,113]
**Glutamine metabolism**			
GLS1 ^6^	BPTES CB-839	Osteosarcoma, pancreatic cancer Multiple cancers	Preclinical [114,115] Clinical phase I/II [32]

^1^ 2-DG, 2-deoxyglucose; ^2^ PFKFB3, fructose-2,6-biphosphatase 3; ^3^ 3PO, 3-(3-pyridinyl)-1-(4-pyridynyl)-2-propen-1-one; ^4^ PDK1, pyruvate dehydrogenase kinase 1; ^5^ CPT1, carnitine palmitoyltransferase 1; ^6^ GLS1, glutaminase 1.

## Data Availability

Data sharing not applicable.

## Abbreviations

The following Abbreviations are used in the manuscript:

3PG	3-phosphoglyceric acid
3PO	3-(3-pyridinyl)-1-(4-pyridynyl)-2-propen-1-one
AD	aldose reductase
ALDOA/C	aldolase A/aldolase C
AMPK	AMP-activated protein kinase
ATP	adenosine triphosphate
CPT1A	carnitine palmitoyltransferase 1A
DAG	diacylglycerol
DHAP	dihydroxyacetone phosphate
ECFC	endothelial colony-forming cell
ECs	endothelial cells
ENO1	enolase 1
F6P	fructose 6-phosphate
FABP	fatty acid-binding protein
FAO	fatty acid oxidation
FASN	fatty acid synthase
FAT	fatty acid translocase
FATP	fatty acid transport protein
G3P	glyceraldehyde 3-phosphate
G6P	glucose-6-phosphate
GAPDH	glyceraldehyde 3-phosphate dehydrogenase
GLS1	glutaminase 1
GLUT1/3	glucose transporter 1/3
GPI	glucose-6-phosphate isomerase
HIF-1α	hypoxia-inducible factor 1 alpha
HK1/2	hexokinase 1/2
HMGB1	high-mobility group box 1
HUVECs	human umbilical vein endothelial cells
IRS1/2	insulin receptor substrate 1/2
LDHA/LDHB	lactate dehydrogenase A/lactate dehydrogenase B
MCT1/4	monocarboxylate transporter 1/4
mTOR	mammalian target of rapamycin
NAD	nicotinamide adenine dinucleotide
NADPH	nicotinamide adenine dinucleotide phosphate
OXPHOS	oxidative phosphorylation
PAD	peripheral artery disease
PDK1	pyruvate dehydrogenase kinase 1
PFKFB3	6-phosphofructo-2-kinase/fructose-2,6-biphosphatase 3
PFK1	phosphofructokinase 1
PGK	phosphoglycerate kinase
PHD	prolyl hydroxylase domain protein
PKC	protein kinase C
PKM2	pyruvate kinase M2
PPARγ	peroxisome proliferator-activated receptor gamma
PPARα	peroxisome proliferator-activated receptor alpha
ROS	reactive oxygen species
SDH	sorbitol dehydrogenase
SIRT1	sirtuin 1
TECs	tumor endothelial cells
TFEB	transcription factor EB
